# Association of Non-Transfusion-Related Admission Hypocalcaemia With Haemodynamic Instability in Paediatric Major Trauma: A Retrospective Single-Centre Pilot Study

**DOI:** 10.7759/cureus.64983

**Published:** 2024-07-20

**Authors:** Owen Hibberd, Ed Barnard, Matthew Ellington, Tim Harris, Stephen H Thomas

**Affiliations:** 1 Blizard Institute, Queen Mary University London, London, GBR; 2 Emergency and Urgent Care Research in Cambridge (EURECA) PACE Section, Department of Medicine, Cambridge University, Cambridge, GBR; 3 Academic Department of Military Emergency Medicine, Royal Centre for Defence Medicine (Research and Clinical Innovation), Birmingham, GBR; 4 Department of Research, Audit, Innovation, & Development (RAID), East Anglian Air Ambulance, Norwich, GBR; 5 Component Development Laboratory, NHS Blood and Transplant, Cambridge, GBR; 6 Department of Emergency Medicine, Harvard Medical School, Boston, USA

**Keywords:** paediatric surgery, calcium, transfusion, paediatric resuscitation, paediatric trauma

## Abstract

Background

The ‘lethal triad’ of acidosis, hypothermia, and coagulopathy is now considered a diamond of death, with ionised hypocalcaemia (iHypoCa) contributing to cardiovascular decompensation and coagulopathy. iHypoCa may be associated with haemodynamic instability and adverse outcomes in paediatric major trauma patients. However, current data are limited.

The primary aim of this pilot study was to report the association between admission iHypoCa and early hypotension on admission in a cohort of paediatric major trauma patients. Secondary aims include reporting the incidence and differential determinants of iHypoCa and the association with treatment (vasoactive agents, blood transfusion, interventional radiology (IR), or surgery) and adverse outcomes (length of stay, morbidity (Glasgow Outcome Scale), and mortality).

Methods

This pilot study is a retrospective analysis of paediatric major trauma patients (<16 years old) admitted to a major trauma centre (2016-2022). Patients with an admission ionised calcium level obtained before the administration of blood products were included.

Multivariable logistic regression was used to assess the dichotomous endpoint of hypotension (systolic blood pressure of <80 mmHg for <1 year, <85 mmHg for one to five years, <90 mmHg for five to 12 years, <100 mmHg for >12 years) for association with hypocalcaemia and adjusted for other potential variables of interest (age, gender, Injury Severity Score, pre-hospital fluids, and acidosis).

Results

Admission iHypoCa was observed in 8/45 (17.8% (95% confidence interval (CI) 9.3-31.3%)) patients. Other than the adolescent age group (*p *< 0.05), there were no significant differences in the baseline characteristics.

As a pilot study, this was not powered for statistical significance; however, point estimates of the odds of hypotension were almost three times higher for patients with iHypoCa (odds ratio (OR) 2.8 (95% CI 0.4-23.6), *p* = 0.33). An association between iHypoCa and the need for IR/surgery in the first 24 hours of admission was also observed (OR 10.9 (95% CI 1.4-159.4), *p < *0.05).

Conclusion

iHypoCa was observed in approximately one in six paediatric major trauma patients at admission and may be associated with increased odds of requiring IR/surgery. Larger multicentre studies are required to clarify point estimates for treatment requirements and adverse outcomes.

## Introduction

Major trauma is one of the leading causes of morbidity and mortality in children in the United Kingdom (UK) [1]. Haemorrhage is the predominant cause of potentially survivable death in trauma, and a high proportion of these deaths occur pre-hospital [2]. Consequently, the management of traumatic haemorrhage is a UK national research priority in emergency medicine [3]. The ‘lethal triad’ of acidosis, hypothermia, and coagulopathy is now considered a ‘diamond of death’; the fourth component, hypocalcaemia, is critical to trauma resuscitation [4,5]. Calcium homeostasis is required for clotting, cardiac contractility, and vascular tone [4,5]. Therefore, hypocalcaemia can contribute to cardiovascular decompensation and coagulopathy [4,5].

Ionised hypocalcaemia (iHypoCa) in the context of trauma may be secondary to the administration of citrated blood products, a process that is well understood [6,7]. However, recent data suggest that iHypoCa may exist in patients with traumatic haemorrhage before receiving blood, and this may be further exacerbated by the citrate in the transfusion [5,8,9]. Although trauma-induced iHypoCa is likely multifactorial, potential mechanisms include calcium-lactate binding, haemodilution, reduction in parathyroid hormone release, inappropriate renal calcium loss, and intracellular calcium influx in the setting of ischaemia and reperfusion [4,5]. Up to 50% of adult major trauma patients have been observed to have iHypoCa, which is associated with mortality, coagulopathy, shock, and an increase in subsequent blood transfusion requirements [10-14]. Children may be more sensitive to iHypoCa compared to adults due to the different injury mechanisms, patterns of injury, and their maturing haemostatic system [15]. However, paediatric data are limited and heterogeneous [16-20].

The primary aim of this pilot study is to report the association between admission iHypoCa and early hypotension on admission in a cohort of paediatric major trauma patients. Secondary aims include reporting the incidence and differential determinants of iHypoCa and the association with treatment requirements (vasoactive agents, blood product transfusion requirement, interventional radiology (IR), or surgery) and adverse outcomes (length of stay (LOS), morbidity (Glasgow Outcome Scale (GOS)), and mortality).

## Materials and methods

Study design

This study is a retrospective analysis of paediatric major trauma patients admitted to Cambridge University Hospitals NHS Foundation Trust (CUH), a major trauma centre (MTC) in the East of England between August 2016 and March 2022 (see Appendix A). Patients were included if they were <16 years old, had an Injury Severity Score (ISS) ≥ 15, Trauma Audit Research Network (TARN) positive (admission to hospital for three days or longer, intensive care, transfer for further specialist care, death), and had an ionised calcium (iCa) level taken on admission. Patients were excluded if they had a pre-hospital cardiac arrest, received a blood transfusion or exogenous calcium before the first iCa measurement, arrived at the MTC >24 hours after injury, or were treated at another hospital before transfer (secondary transfers or repatriations). The study followed the Strengthening the Reporting of Observational Studies in Epidemiology (STROBE) guideline.

Study procedures

Paediatric major trauma patients were identified from TARN data obtained from the local site Trauma Office records with matched patient data obtained from the electronic medical record (EMR) at the local site (Epic Hyperspace Production®, Epic Systems Corporation, Verona, WI, USA).

Demographics, mechanism of injury, injury time, ISS, need for surgical management, 24-hour and 30-day mortality, functional outcome (GOS score), and hospital and paediatric intensive care unit (PICU) LOS data were obtained from the local Trauma Office records. The presence of hypotension (systolic blood pressure of <80 mmHg for <one year, <85 mmHg for one to five years, <90 mmHg for five to 12 years, <100 mmHg for >12 years; defined dichotomously based upon standard reference values) [21], and details of pre-hospital and hospital treatments (intravenous fluids, blood transfusion, exogenous calcium supplementation and whether this was before or after blood product transfusion, vasoactive agents, and IR/surgery) were obtained from the EMR. In addition, admission blood gas data and physiological observations were extracted from the EMR. Point-of-care levels of iCa were defined dichotomously as iHypoCa (iCa < 1.16 mmol/L) and normocalcaemia (iNormoCa) (iCa ≥ 1.16 mmol/L) to reflect previous literature [20]. Levels of iCa were not adjusted for pH as this risks underestimating iHypoCa and the utility of adjusting this in the clinical setting is unclear [20].

Sample size

As a single-centre pilot study, a consecutive sample of 45 paediatric major trauma patients with iCa results was targeted (Appendix B). This sample size was estimated based on the extrapolation of previous patient numbers in a regional study [22]. The proportioned sample size for a subsequent multicentre study is shown in Appendix C.

Outcomes

The primary outcome of the presence of hypotension (defined dichotomously based upon age-related values) was measured as the lowest recorded systolic blood pressure on emergency department admission (or as close as possible to allow a temporal link with blood gas data).

Secondary outcomes included the need for exogenous calcium, vasoactive medication, transfusion, IR/surgery within the first 24 hours of admission (defined dichotomously), and hospital/PICU LOS. Mortality within 24 hours and 30 days was also measured, as was functional outcome at 30 days (GOS score).

Method of analysis

A password-protected and encrypted Excel spreadsheet (Microsoft Office Professional 2023, V16.71, Microsoft Corporation, Redmond, Washington, USA) was used for processing data, and Prism (Prism 2023, V10.1.0 (264), GraphPad Software, Boston, Massachusetts, USA) was used for the statistical analysis.

Basic demographics, mechanism of injury, and injury data are reported as numbers (percentages) and mean (+/- standard deviation (SD)) or median (interquartile range (IQR)). Normality was assessed using Shapiro-Wilk formal testing and quantile-normal plotting. Normally distributed parameters were compared using Student’s t-test. The Mann-Whitney U test was used for non-normally distributed variables. The χ2 test was used to analyse categorical variables.

Multivariable logistic regression (supplementary appendix) was used to assess the dichotomous endpoint of hypotension for association with iHypoCa. The logistic regression adjusted for other potential variables of interest (age, gender, ISS, pre-hospital fluids, and acidosis (pH <7.35)). Wald and likelihood-ratio testing were used to evaluate different models’ relative performance. Logistic regression models (Appendix D) were evaluated using post-estimation evaluation for calibration (e.g., Hosmer-Lemeshow goodness-of-fit, Cox plotting) and discrimination (e.g., cross-validated area c statistic).

Risk of bias

Previous studies have been affected by selection bias in their use of trauma team activation to include patients; this study’s use of TARN criteria aims to mitigate this by capturing all relevant paediatric major trauma patients [16-18,20]. Availability and recall bias are also relevant for documenting the handover of treatments given pre-hospital. To mitigate these biases, the original scanned pre-hospital documentation was reviewed and checked against data in the EMR.

Ethics

A formal ethical review was obtained from the UK Health Research Authority Research Ethics Committee (REC), with reference number 23/PR/0876.

Patient and public involvement

No patient and public involvement activities were undertaken for this study.

## Results

Participants

**Figure 1:**
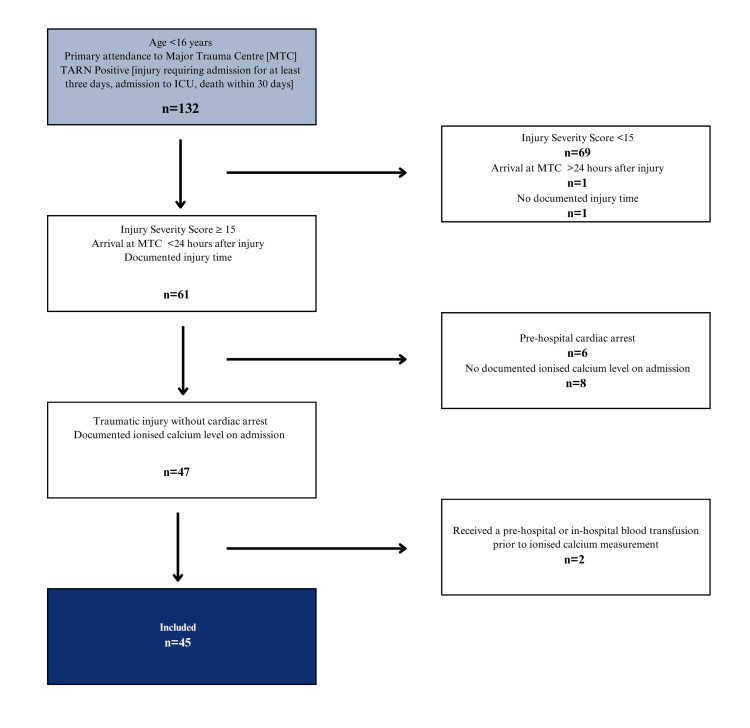
Patient flow diagram demonstrating the inclusion of 45 paediatric major trauma patients with an admission ionised calcium level

All paediatric major trauma patients with an admission iCa level were included in the analysis, which included 45 patients (Figure 1).

Descriptive data

The median age was 10.8 (IQR 5.6-14.9) years, and 25/45 (55.5%) of the patients were male. The median ISS was 25 (IQR 20-35), and most patients had a blunt mechanism of injury (44/45 (97.8%)).

Out of the 45 patients included in this study, eight (17.8% (95% CI 9.3-31.3%)) were observed to have admission iHypoCa (Table 1).

**Table 1:** Demographics and clinical characteristics for 45 paediatric major trauma patients in relation to ionised calcium level taken on emergency department admission Hypocalcaemia = ionised calcium <1.16 mmol/L; normocalcaemia = ionised calcium ≥ 1.16 mmol/L; infants = 0-1 years; toddlers = 2-3 years; preschool = 4-5 years; school = 6-12 years; adolescents = 12-16 years; ISS = Injury Severity Score; IQR = interquartile range

	Total (n = 45)	Normocalcaemia (n = 37)	Hypocalcaemia (n = 8)	P-value
					Age in years, median [IQR]	10.8 [5.6-14.9]	10.1 [5.6-14.1]	14.7 [12.8-15]	0.09
Infants (0-1 years), n (%)	1.0 (2.2%)	1.0 (2.7%)	0.0 (0.0%)	0.99
Toddlers (2-3 years), n (%)	6.0 (13.3%)	5.0 (13.5%)	1.0 (12.5%)	0.99
Preschool (4-5 years), n (%)	7.0 (15.6%)	7.0 (19.0%)	0.0 (0.0%)	0.32
School (6-12 years), n (%)	12.0 (26.7%)	12.0 (32.4%)	0.0 (0.0%)	0.08
Adolescent (12-16 years), n (%)	19.0 (42.2%)	12.0 (32.4%)	7.0 (87.5%)	<0.05
					Male gender, n (%)	25.0 (55.5%)	21.0 (56.7%)	4.0 (50.0%)	0.73
ISS, median [IQR]	25.0 [20.0–33.0]	25.0 [19.0–34.0]	25.0 [22.0–26.0]	0.76					
					Abdomen, most injured region, n (%)	5.0 (11.1%)	4.0 (10.8%)	1.0 (12.5%)	0.99
Chest, most injured region, n (%)	4.0 (8.9%)	4.0 (10.8%)	0.0 (0.0%)	0.99
Head, most injured region, n (%)	25.0 (55.5%)	21.0 (56.8%)	4.0 (50.0%)	0.99
Limbs, most injured region, n (%)	3.0 (6.7%)	3.0 (8.1%)	0.0 (0.0%)	0.99
Multiple most injured regions, n (%)	7.0 (15.6%)	4.0 (10.8%)	3.0 (37.5%)	0.09
Spine, most injured region, n (%)	1.0 (2.2%)	1.0 (2.7%)	0.0 (0.0%)	0.99
Blunt trauma, n (%)	44.0 (97.7%)	36.0 (97.3%)	8.0 (100.0%)	0.63
					Injury time to ED arrival (minutes), median [IQR]	110.0 [94.0–128.0]	106.0 [94.0–126.0]	120.0 [99.5-142.5]	0.42
ED arrival to calcium measurement (minutes), median [IQR]	11.0 [5.0–25.0]	10.0 [5.0–20.0]	28.5 [9.75-62.5]	0.17

Other than the adolescent age group (p <0.05), there were no statistically significant differences in the demographics and baseline clinical characteristics between the iHypoCa and iNormoCa groups.

Patients arrived at a median of 110.0 (IQR 94.0-128.0) minutes after their injury and had an iCa level measured at 11.0 (IQR 5.0-25.0) minutes after hospital arrival.

Primary and secondary outcomes

Overall, hypotension was observed in 11/45 (24.4% (95% CI 14.2-38.7%)) of the patients; hypotension proportions did not differ between the iHypoCa and NormoCa groups.

There were no differences between groups in markers of perfusion (pH, lactate) or coagulation (PT, APTT, and fibrinogen). In addition, there were no differences between groups for the adverse outcomes of mortality, morbidity (GOS ≤4), or PICU and hospital LOS (Table 2).

**Table 2:** Laboratory abnormalities and adverse outcomes for 45 paediatric major trauma patients in relation to ionised calcium level taken on emergency department admission Hypocalcaemia = ionised calcium < 1.16 mmol/L; normocalcaemia = ionised calcium ≥ 1.16 mmol/L; acidosis = pH < 7.32; hyperkalaemia = potassium > 5.5 mmol/L; hyperlactatemia ≥ 2.0 mmol/L;  raised PT ≥ 12.6 seconds for <one year, >13.4 seconds for one to five years, >14.6 seconds for six to 10 years, >14.1 seconds for ≥11 years; raised APTT ≥ 40.7 seconds for <one year, >39.2 seconds for one to five years, >38.7 seconds for six to 10 years, >38.4 seconds for ≥11 years; low fibrinogen ≤ 1.41 g/L for <one year, <1.64 g/L for one to five years, <1.71 g/L for six to 10 years, <1.68 g/L for ≥11 years; hypotension = systolic blood pressure of <80 mmHg for <1 year, <85 mmHg for one to five years, <90 mmHg for five to 12 years, <100 mmHg for >12 years; SIPA = Shock Index, Paediatric Age-Adjusted; elevated SIPA = SIPA >1.2 for zero to six years, >1 for seven to 12 yrs, >0.9 for >12 years; GOS = Glasgow Outcome Scale; LOS = length of stay; PICU = Paediatric Intensive Care Unit; IQR = interquartile range

	Total (n = 45)	Normocalcaemia (n = 37)	Hypocalcaemia (n = 8)	p-value
					Acidosis, n (%)	22.0 (48.8%)	19.0 (51.4%)	3.0 (37.5%)	0.69
Hyperkalaemia, n (%)	0.0 (0.0%)	0.0 (0.0%)	0.0 (0.0%)	N/A					
					Hyperlactataemia, n (%)	35.0 (77.7%)	28.0 (75.6%)	7.0 (87.5%)	0.66
Raised PT, n (%)	19.0 (42.2%)	18.0 (48.6%)	1.0 (12.5%)	0.11					
Raised APTT, n (%)	3.0 (6.7%)	2.0 (5.4%)	1.0 (12.5%)	0.45
Low fibrinogen, n (%)	5.0 (11.1%)	4.0 (10.8%)	1.0 (12.5%)	0.99
					Hypotension, n (%)	11.0 (24.4%)	8.0 (21.6%)	3.0 (37.5%)	0.38
Elevated SIPA, n (%)	13.0 (28.8%)	10.0 (27.0%)	3.0 (37.5%)	0.67					
					24-hour mortality, n (%)	0.0 (0.0%)	0.0 (0.0%)	0.0 (0.0%)	N/A
30-day mortality, n (%)	1.0 (2.2%)	1.0 (2.7%)	0.0 (0.0%)	N/A					
GOS ≤ 4, n (%)	9.0 (20.0%)	8.0 (21.6%)	1.0 (12.5%)	0.99
					Hospital LOS (days), median [IQR]	9.0 [6.0–20.0]	9.0 [6.0–20.0]	6.5 [5.8-20.5]	0.69
PICU LOS (days), median [IQR]	2.0 [1.0–6.0]	2.0 [1.0–6.0]	1.0 [1.0-4.8]	0.84					

Eight of 45 (17.8% (95% CI 9.3-31.3%)) patients received pre-hospital fluids, whilst 41/45 (91.1% (95% CI 79.3-96.5%)) received intravenous fluids ≤24 hours of admission; proportions did not differ between the iHypoCa and iNormoCa groups. Six of 45 (13.3% (95% CI 6.3-26.2%)) received blood in the first 24 hours, and this was comparable between groups (Table 3). Two of 45 (4.4%) patients received exogenous calcium administration after blood product transfusion; both of these patients were in the iHypoCa group, and neither had severe iHypoCa (<1.0 mmol/L) at the time of the first blood gas measurement.

**Table 3:** Pre-hospital treatments and treatments within the first 24 hours for 45 paediatric major trauma patients, dichotomised by the ionised calcium level on arrival to the emergency department Hypocalcaemia = ionised calcium <1.16 mmol/L; normocalcaemia = ionised calcium ≥ 1.16 mmol/L; IQR = interquartile range; SD = standard deviation; IR = interventional radiology

	Total (n = 45)	Normocalcaemia (n = 37)	Hypocalcaemia (n = 8)	p-value
					Pre-hospital fluid, n (%)	8.0 (17.8%)	7.0 (18.9%)	1.0 (12.5%)	0.99
Pre-hospital fluid mls/kg, mean (±SD)	6.7 (±2.0)	7.1 (±1.9)	6.8 (±0.0)	N/A					
					Fluid in the first 24 hours of admission, n (%)	41.0 (91.1%)	33.0 (89.1%)	8.0 (100.0%)	0.99
Fluid mls/kg in the first 24 hours of admission, median [IQR]	45.0 [37.2-60.9]	42.8 [32.0-61.2]	46.0 [43.3-57.1]	0.59					
					Blood transfusion in the first 24 hours of admission, n (%)	6.0 (13.3%)	4.0 (10.8%)	2.0 (25.0%)	0.28
Blood transfusion mls/kg in the first 24 hours of admission, mean (±SD)	29.3 (±19.5)	24.6 (±5.2)	39.5 (±30.4)	0.46
Calcium administration in the first 24 hours of admission, n (%)	2.0 (4.4%)	0.0 (0.0%)	2.0 (25.0%)	<0.05
Vasoactive medication administration in the first 24 hours of admission, n (%)	9.0 (20.0%)	7.0 (18.9%)	2.0 (25.0%)	0.64
					IR or surgery in the first 24 hours of admission, n (%)	33.0 (73.3%)	27.0 (72.9%)	6.0 (75.0%)	0.91

Logistic regression

None of the differential determinants included in the logistic regression model were statistically significant (age, gender, ISS, pre-hospital fluids, and acidosis) (supplementary appendix). These differential determinants were subsequently included in a multivariable logistic regression model for adverse outcomes (Table 4). 

**Table 4:** Results of the logistic regression for the adverse outcomes associated with admission ionised hypocalcaemia for the 45 paediatric major trauma patients Hypotension = systolic blood pressure of <80 mmHg for <one year, <85 mmHg for one to five years, <90 mmHg for five to 12 years, <100 mmHg for >12 years; elevated SIPA = Shock Index, Paediatric Age-Adjusted, SIPA >1.2 for zero to six years, >1 for seven to 12 years, >0.9 for >12 years;  IR = interventional radiology; GOS = Glasgow Outcome Scale

	Odds ratio	95% confidence interval	p-value
Hypotension	2.8	0.4-23.6	0.33
				Elevated SIPA	3.1	0.1-81.0	0.46
Need for blood transfusion in the first 24 hours	11.4	0.7-387.1	0.11				
				Need for vasoactive medication in the first 24 hours	2.5	0.2-30.2	0.43
Need for IR/surgery in the first 24 hours	10.9	1.4-159.4	<0.05				
				GOS ≤ 4	2.5	0.1-47.2	0.54

This multivariable logistic regression model demonstrated point estimates toward increased odds of all adverse outcomes for the iHypoCa group. However, the only statistically significant adverse outcome was the need for IR/surgery in the first 24 hours (OR 10.9 (95% CI 1.4-159.4) p ≤ 0.05). Mortality was unable to be included in the model due to the low mortality rate in this cohort, 1/45 (2.2% (95% CI 0.1-11.6%)).

## Discussion

In this pilot study, admission iHypoCa was observed to be present in approximately one in six patients and was more prevalent amongst adolescents. A multivariable logistic regression model for adverse outcomes demonstrated a point estimate in the direction of iHypoCa patients having two to three times the odds of haemodynamic instability compared to iNormoCa patients; however, this was underpowered due to the small sample. In the first 24 hours, a 10-fold increased odds of IR/surgery were observed. There were also point estimates in the direction of iHypoCa being associated with all other adverse outcomes.

Incidence and differential determinants

In this study, the incidence of iHypoCa was similar to the incidence of 112/710 (15.8%) reported amongst paediatric major trauma patients in a systematic review and meta-analysis [20]. However, the systematic review observed a wide range of definitions of iHypoCa (<1.00 mmol/l to <1.16 mmol/l) and variability in incidence (5.3-46.5%) [20]. Conversely, the highest incidence of 66/142 (46.5%) was observed amongst the cohort described by Ciaraglia et al., for whom the definition of iHypoCa was the lowest [17,20]. This may be a result of the high proportion of penetrating injuries (38/142 (26.7%)) observed amongst the patients in the study reported by Ciaraglia et al., although this was not statistically significant between the iHypoCa and iNormoCa groups [17]. Studies among adult major trauma patients have also observed a wide range of definitions and incidence [5,10,13]. Although a systematic review involving adult major trauma patients reported the incidence to range between 23.0% and 56.2%, two recent retrospective cohort studies comprising a larger sample size than meta-analysed data from the systematic review have also reported a variable incidence: 869/1,981 (43.9%) in an American single-centre study by Ciaraglia et al. and 3,982/30,183 (13.2%) in a European multicentre study by Helsloot et al. [5,10,13]. Amongst paediatric major trauma patients, larger multicentre studies are required to assess the most appropriate cut-offs and the true incidence of admission iHypoCa.

In this single-centre study, no statistically significant difference was seen for gender, trauma mechanism, most injured region of the body, ISS, or administration of pre-hospital fluids; however, this should be interpreted with caution due to the small sample size. Age was observed to be a significant differential determinant, with a greater incidence of iHypoCa observed amongst adolescent age groups in both this single-centre study, and in the systematic review, an age group where physiology and response to trauma injury may be more like adult patients [20]. Further research is required to determine whether iHypoCa in paediatric trauma should be considered different to the adult trauma population and whether there are relevant age-related cut-offs.

Studies amongst adults have shown injury severity to be significantly associated with iHypoCa [5,10,11,13]. There are also a few studies, which indicate that penetrating injury and blast injury may be of significance [6,13,23]. Amongst paediatric patients, a larger dataset is required to demonstrate any association with the mechanism of injury, with such data having great utility in emergency medicine with regard to trauma pre-alerts and subsequent preparation. Timing of iHypoCa is another important differential determinant, which may be useful in the pre-hospital and early management of trauma. This single-centre study is novel in reporting the timing of iCa measurement with regard to injury and hospital arrival times; although no significant difference was seen, this may be a result of the small sample size and the operational efficiency of the regional trauma network [22]. Further work may be useful in exploring at what time iHypoCa occurs and whether this occurs at the point of injury.

Haemodynamic instability

Only two studies involving paediatric major trauma patients have explored the association between iHypoCa and haemodynamic instability, both looking at the Shock Index, Paediatric Age-Adjusted (SIPA) [17,18]. Ciaraglia et al. observed 28/66 (42.4%) of iHypoCa patients to be haemodynamically unstable, and similar to this single-centre study, this was associated with a threefold increased odds of haemodynamic instability (OR 3.6 (95%CI 1.7-7.7)) [17]. By contrast, Epstein et al. observed 6/24 (25.0%) of iHypoCa patients to be haemodynamically unstable and that this was not associated with increased odds of haemodynamic instability (OR 0.9 (95%CI 0.3-2.3)) [18]. Among adult major trauma patients, both hypotension and a significantly worse shock index amongst iHypoCa (iCa < 1.0 mmol/l) have been observed across studies [5,10,12,13]. All these studies had a lower iHypoCa cut-off than in this single-centre study. The aetiology and degree of tachycardia in paediatric patients can also be variable [24]. Therefore, larger multicentre studies would benefit from exploring whether the observed point estimate towards haemodynamic instability is statistically significant with a larger sample size and at what cut-off for iHypoCa haemodynamic instability is seen.

Laboratory abnormalities

In this single-centre study, no significant differences were seen between the iHypoCa and iNormoCa groups for the markers of physiological derangement, such as pH and lactate or markers of clotting. This is similar to results from the paediatric systematic review and meta-analysis [20]. By contrast, adult major trauma patients with iHypoCa have been observed to be at greater risk for acidosis, hyperlactatemia, and raised base deficit, potentially reflecting impaired perfusion and ischaemic injury [10,13]. This is physiologically significant as it further potentiates iHypoCa due to pH-dependent calcium-lactate binding and stimulation of parathyroid hormone secretion, and thus also affects the other components of the 'diamond of death' [4]. The difference in results between paediatric and adult studies may reflect the paucity of paediatric data compared to adult data or may suggest an underlying physiological difference based on age group, which requires further exploration.

Studies of adult major trauma patients have shown markers of clotting to be heterogeneously reported across studies, and iHypoCa has been observed to be associated with coagulopathy, with studies reporting significantly increased INR, PT, and APTT [5,9,10,12,13]. Mechanistically, calcium is an essential co-factor in the clotting cascade and is important for platelet activation and aggregation [25]. Minimal research has included iHypoCa major trauma patients with thromboelastography (TEG) or rotational thromboelastometry (ROTEM) measurement, which may be due to this not being widely used in clinical practice [26]. Future studies would benefit from incorporating viscoelastic measurements to appreciate the relationship between iHypoCa and coagulopathy better.

Treatments received

Although not powered for statistical significance, this single-centre pilot study suggested a point estimate towards increased odds of blood transfusion requirements and receipt of vasoactive medications. By contrast, other studies in both paediatric and adult trauma populations have demonstrated that iHypoCa patients have a statistically significant increased transfusion requirement [5,9,13,18,20] and requirement for vasoactive medications [13]. This may be important when planning treatment and resuscitation strategies. Current guidelines recommend maintaining normal iCa levels in the bleeding trauma patient [27]. In addition, studies have demonstrated that iHypoCa pre-transfusion can be significantly worsened following the administration of even a single unit of citrated blood products [6]. Therefore, recognition and early treatment of iHypoCa is prudent.

This single-centre study is novel in reporting the need for IR/surgery. The odds of requiring IR/surgery in the first 24 hours of admission in the iHypoCa group were nearly 11-fold, and this was statistically significant. This is again significant in considering which patients may require more aggressive treatment and also has significance when considering the wider physical and psychological morbidity that occurs in children undergoing such treatments [28]. Since this was significant in the multivariate analysis but not univariate analysis, there is a suggestion that other differential determinants, such as injury severity, may be relevant, and this would benefit from further exploration in a larger multicentre study.

Adverse outcomes

Amongst paediatric patients, the functional outcome (GOS score) may be a more meaningful outcome than mortality due to the relatively low overall mortality in this cohort [29]. This is shown when comparing mortality outcomes between paediatric and adult studies, with paediatric studies not demonstrating a mortality difference, whilst adult studies showed a difference across studies [5,10,12,13,14]. However, amongst adult major trauma patients, Helsloot et al. demonstrated that iCa levels have a parabolic relationship with adverse outcomes, with both iHypoCa and hypercalcaemia being associated with poor outcomes [13]. Moreover, a systematic review of empirical calcium administration in cardiac arrest suggested that this may be associated with harm [30]. As such, the effect of calcium administration is unclear and empirical administration is not recommended. Further studies exploring whether exogenous administration of calcium for paediatric patients with admission iHypoCa improves outcomes are recommended.

Limitations

The small sample size and single-centre retrospective design limited this study as it was underpowered to detect differences in the primary outcome; however, point estimates were useful in hypothesis generation for future multicentre studies. As a reflection of the small sample size, there was also a small number of penetrating injuries, which limits generalisability, and mortality was so low that this was not able to be formally analysed. These may have been important outcomes to include in a multivariate analysis. However, as a single-centre pilot study, this methodology is appropriate, and these limitations can be overcome by future multicentre expansion of the study and accordant adjustment of models of analysis. Given the large number of explanatory variables relative to the number of occurrences of the objective variable, the accuracy of the logistic regression model may have been affected. The multicentre expansion of this study would benefit from evaluating the AIC to optimise the model and evaluate its accuracy.

Bias associated with the retrospective design was mitigated by a manual review of the scanned and electronic medical records and quality checking of data extraction by a second author (supplementary appendix). However, although there was a detailed review of the records, extraction of the most injured regions may have missed neck injuries and/or parathyroid injuries, which could directly influence iCa levels. In addition, details on the type of surgery/IR required were not explored. Despite such limitations, this study is novel in considering a number of potential confounders related to pre-hospital treatments and timing of measurement that have not been considered across previous paediatric studies and may be relevant differential determinants of ionised hypocalcaemia [23]. 

## Conclusions

Non-transfusion-related admission iHypoCa was observed in approximately one in six paediatric major trauma patients at admission and was more prevalent amongst adolescents. This may be associated with an increased need for IR/surgery in the first 24 hours. Larger multicentre studies are required to clarify point estimates for treatment requirements and adverse outcomes.

## Figures and Tables

**Figure 2:**
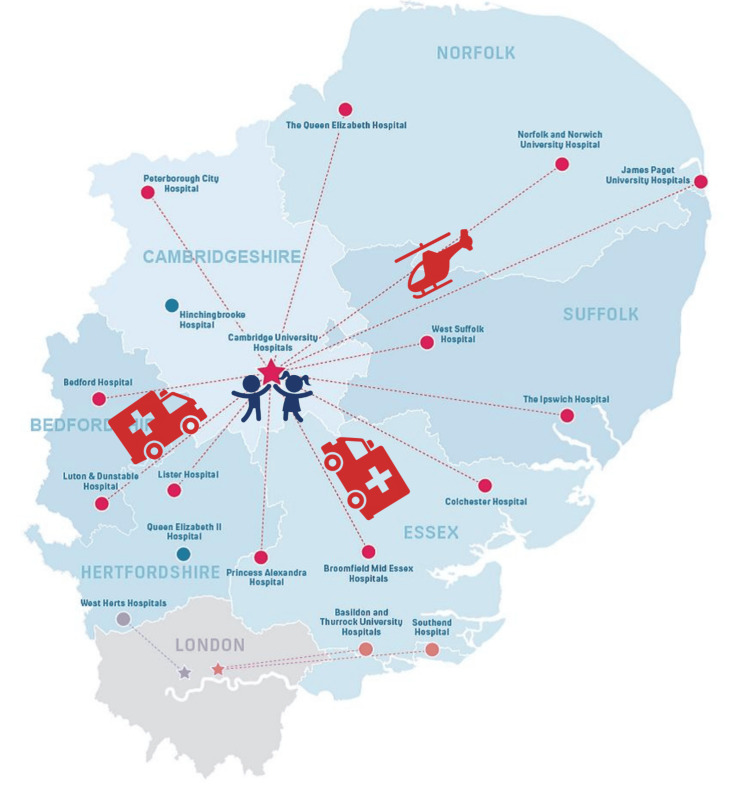
The East of England Trauma Network Figure adapted from the East of England Trauma Network: TEMPO, Trauma East Manual of Procedures and Operations, Edition 2, East of England Trauma Network, 2022.

**Figure 3:**
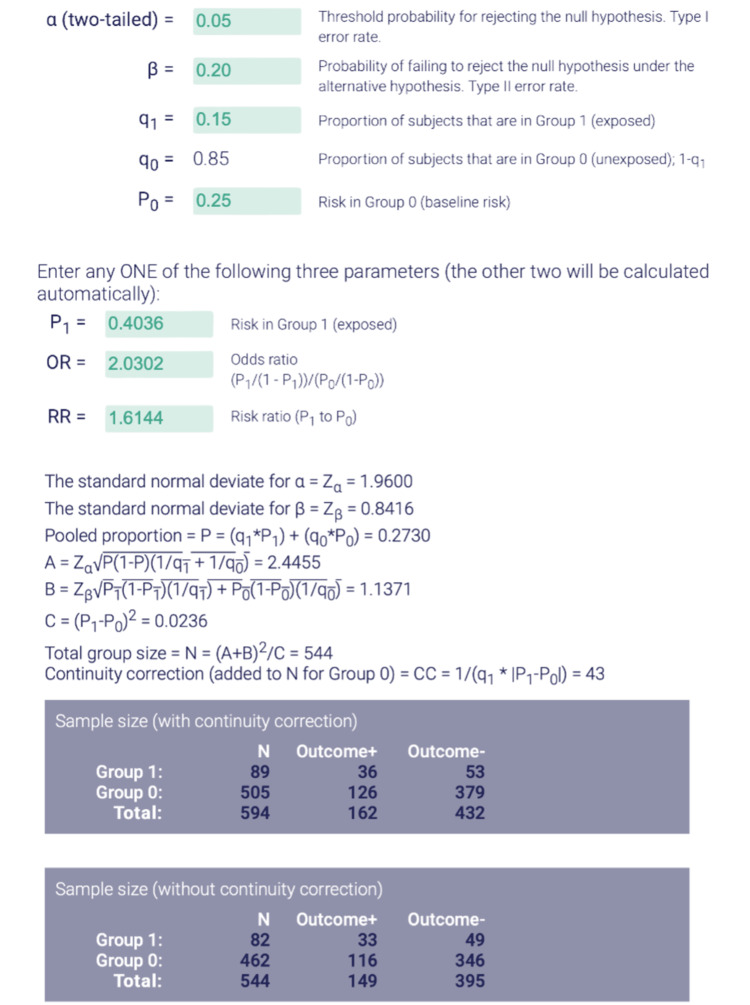
Proportioned sample size calculation Sample size calculated on https://sample-size.net/sample-size-proportions/ Sample size calculated from the results of Hibberd O, Price J, Thomas SH, et al. The incidence of admission ionised hypocalcaemia in paediatric major trauma-A systematic review and meta-analysis. PLoS One. 2024;19(5):e0303109. Published 2024 May 28. doi:10.1371/journal.pone.0303109

## Appendices

Appendix A

The East of England Trauma Network

This pilot study was undertaken at Cambridge University Hospitals NHS Foundation Trust (CUH) major trauma centre (MTC) for the East of England Trauma Network, covering a population of 6.3 million people over 20,000 km^2^. This is an inclusive trauma network that covers the geographical areas of Cambridgeshire, Bedfordshire, Essex, Hertfordshire, Norfolk, and Suffolk. All areas have been operational since 2012 (Figure 2).

Appendix B

Data Monitoring

For monitoring and validation purposes, a second data collector independently recollected 10% of the data. These data were compared to the collected data, and any discrepancies prompted a re-review of the data. There were no data discrepancies requiring re-review.

Appendix C

Proportioned Sample Size Calculation

The proportioned sample size calculation for a subsequent multicentre study is shown below. The calculation aims for a consecutive sample of approximately 600 paediatric major trauma patients (Figure 3).

The time frame for data collection in this pilot was limited by the availability of data from the Trauma and Audit Research Network (TARN) on a national level.

Appendix D

Logistic Regression Modelling

Logistic regression modelling for the differential determinants of admission ionised hypocalcaemia is shown in Table 5.

**Table 5:** Logistic regression for the differential determinants of admission ionised hypocalcaemia for 45 paediatric major trauma patients

Parameter estimates	Variable	Estimate	Standard error	95% CI (profile likelihood)	
β0	Intercept	-2.798	1.917	-7.163 to 0.7230	
β1	Age	0.22	0.1185	0.02065 to 0.5067	
β2	Gender	-0.4135	0.9077	-2.268 to 1.373	
β3	ISS	-0.01796	0.0533	-0.1412 to 0.07849	
β4	Pre-hospital fluids	-0.6728	1.386	-3.959 to 1.908	
β5	Acidosis (pH < 7.32)	-0.8944	0.9559	-2.916 to 0.9167	
Odds ratios	Variable	Estimate	95% CI (profile likelihood)		
β0	Intercept	0.06095	0.0007744 to 2.061		
β1	Age	1.246	1.021 to 1.660		
β2	Gender	0.6613	0.1036 to 3.947		
β3	ISS	0.9822	0.8683 to 1.082		
β4	Pre-hospital fluids	0.5103	0.01908 to 6.739		
β5	Acidosis (pH < 7.32)	0.4089	0.05417 to 2.501		
Sig. diff. than zero?	Variable	|Z|	P-value	P-value summary	
β0	Intercept	1.459	0.1445	ns	
β1	Age	1.856	0.0634	ns	
β2	Gender	0.4556	0.6487	ns	
β3	ISS	0.3369	0.7362	ns	
β4	Pre-hospital fluids	0.4856	0.6273	ns	
β5	Acidosis (pH < 7.32)	0.9356	0.3495	ns	
Model diagnostics	Degrees of Freedom	AICc			
Intercept-only model	44	44.21			
Selected model	39	50.25			
Area under the ROC curve					
Area	0.7365				
Std. error	0.09696				
95% confidence interval	0.5464 to 0.9265				
P-value	0.0377				
Classification table	Predicted 0	Predicted 1	Total	% Correctly classified	
Observed 0	37	0	37	100	
Observed 1	6	2	8	25	
Total	43	2	45	86.67	
Negative predictive power (%)	86.05				
Positive predictive power (%)	100				
Classification cutoff	0.5				
Pseudo R squared					
Tjur's R squared	0.1436				
Cox-Snell's R squared	0.1265				
Hypothesis tests	Statistic	P-value	Null hypothesis	Reject Null Hypothesis?	P-value summary
Hosmer-Lemeshow	5.875	0.6612	Selected model is correct	No	ns
Log-likelihood ratio (G squared)	6.086	0.298	Simpler (intercept-only) model is correct	No	ns

The logistic regression modelling for the adverse outcome of hypotension associated with admission ionised hypocalcaemia is shown in Table 6.

**Table 6:** Logistic regression for the adverse outcome of hypotension associated with admission ionised hypocalcaemia for 45 paediatric major trauma patients

Parameter estimates	Variable	Estimate	Standard error	95% CI (profile likelihood)	
β0	Intercept	-2.543	1.903	-6.867 to 1.002	
β1	Presence of hypotension in first 24 hours	1.02	1.038	-1.045 to 3.160	
β2	Age	0.2097	0.1183	0.01070 to 0.4977	
β3	Gender	-0.2757	0.9357	-2.167 to 1.594	
β4	ISS	-0.04215	0.06073	-0.1828 to 0.06635	
β5	Pre-hospital fluids	-0.214	1.495	-3.625 to 2.670	
β6	Acidosis (pH < 7.32)	-0.7798	0.9953	-2.867 to 1.138	
Odds ratios	Variable	Estimate	95% CI (profile likelihood)		
β0	Intercept	0.07867	0.001042 to 2.723		
β1	Presence of hypotension in first 24 hours	2.773	0.3518 to 23.56		
β2	Age	1.233	1.011 to 1.645		
β3	Gender	0.7591	0.1145 to 4.925		
β4	ISS	0.9587	0.8329 to 1.069		
β5	Pre-hospital fluids	0.8074	0.02664 to 14.43		
β6	Acidosis (pH < 7.32)	0.4585	0.05687 to 3.121		
Sig. diff. than zero?	Variable	|Z|	P-value	P-value summary	
β0	Intercept	1.336	0.1815	ns	
β1	Presence of hypotension in first 24 hours	0.9829	0.3257	ns	
β2	Age	1.773	0.0763	ns	
β3	Gender	0.2946	0.7683	ns	
β4	ISS	0.6941	0.4876	ns	
β5	Pre-hospital fluids	0.1431	0.8862	ns	
β6	Acidosis (pH < 7.32)	0.7835	0.4333	ns	
Model diagnostics	Degrees of Freedom	AICc			
Intercept-only model	44	44.21			
Selected model	38	52.09			
Multicollinearity	Variable	VIF	R2 with other variables		
β0	Intercept				
β1	Presence of hypotension in first 24 hours	1.204	0.1697		
β2	Age	1.167	0.1431		
β3	Gender	1.234	0.1895		
β4	ISS	1.823	0.4514		
β5	Pre-hospital fluids	1.647	0.3929		
β6	Acidosis (pH < 7.32)	1.193	0.162		
Area under the ROC curve					
Area	0.7838				
Std. error	0.07695				
95% confidence interval	0.6330 to 0.9346				
P-value	0.0126				
Classification table	Predicted 0	Predicted 1	Total	% Correctly classified	
Observed 0	37	0	37	100	
Observed 1	7	1	8	12.5	
Total	44	1	45	84.44	
Negative predictive power (%)	84.09				
Positive predictive power (%)	100				
Classification cutoff	0.5				
Pseudo R squared					
Tjur's R squared	0.1548				
Cox-Snell's R squared	0.1451				
Hypothesis tests	Statistic	P-value	Null hypothesis	Reject null hypothesis?	P-value summary
Hosmer-Lemeshow	8.108	0.423	Selected model is correct	No	ns
Log-likelihood ratio (G squared)	7.055	0.3158	Simpler (intercept-only) model is correct	No	ns

The logistic regression modelling for the adverse outcome of elevated shock index associated with admission ionised hypocalcaemia is shown in Table 7.

**Table 7:** Logistic regression for the adverse outcome of elevated shock index (paediatric age-adjusted) associated with admission ionised hypocalcaemia for 45 paediatric major trauma patients

Parameter estimates	Variable	Estimate	Standard error	95% CI (profile likelihood)	
β0	Intercept	-3.171	2.061	-7.939 to 0.5423	
β1	Elevated SIPA	1.144	1.551	-2.265 to 4.394	
β2	Age	0.264	0.1409	0.03286 to 0.6068	
β3	Gender	-0.3476	0.9175	-2.216 to 1.461	
β4	ISS	-0.02882	0.05662	-0.1587 to 0.07256	
β5	Pre-hospital fluids	-0.6845	1.436	-4.073 to 1.992	
β6	Acidosis (pH < 7.32)	-1.04	0.9792	-3.105 to 0.8159	
Odds ratios	Variable	Estimate	95% CI (profile likelihood)		
β0	Intercept	0.04195	0.0003566 to 1.720		
β1	Elevated SIPA	3.141	0.1039 to 81.00		
β2	Age	1.302	1.033 to 1.835		
β3	Gender	0.7064	0.1091 to 4.310		
β4	ISS	0.9716	0.8532 to 1.075		
β5	Pre-hospital fluids	0.5044	0.01703 to 7.333		
β6	Acidosis (pH < 7.32)	0.3534	0.04482 to 2.261		
Sig. diff. than zero?	Variable	|Z|	P-value	P-value summary	
β0	Intercept	1.539	0.1238	ns	
β1	Elevated SIPA	0.738	0.4605	ns	
β2	Age	1.874	0.061	ns	
β3	Gender	0.3788	0.7048	ns	
β4	ISS	0.5091	0.6107	ns	
β5	Pre-hospital fluids	0.4766	0.6336	ns	
β6	Acidosis (pH < 7.32)	1.062	0.2881	ns	
Model diagnostics	Degrees of Freedom	AICc			
Intercept-only model	44	44.21			
Selected model	38	52.53			
Multicollinearity	Variable	VIF	R2 with other variables		
β0	Intercept				
β1	Elevated SIPA	1.257	0.2041		
β2	Age	1.286	0.2224		
β3	Gender	1.234	0.1899		
β4	ISS	1.676	0.4034		
β5	Pre-hospital fluids	1.514	0.3394		
β6	Acidosis (pH < 7.32)	1.166	0.1421		
Area under the ROC curve					
Area	0.7601				
Std. error	0.08294				
95% confidence interval	0.5976 to 0.9227				
P-value	0.0223				
Classification table	Predicted 0	Predicted 1	Total	% Correctly classified	
Observed 0	37	0	37	100	
Observed 1	6	2	8	25	
Total	43	2	45	86.67	
Negative predictive power (%)	86.05				
Positive predictive power (%)	100				
Classification cutoff	0.5				
Pseudo R squared					
Tjur's R squared	0.1469				
Cox-Snell's R squared	0.1367				
Hypothesis tests	Statistic	P-value	Null hypothesis	Reject null hypothesis?	P-value summary
Hosmer-Lemeshow	2.764	0.9483	Selected model is correct	No	ns
Log-likelihood ratio (G squared)	6.615	0.358	Simpler (intercept-only) model is correct	No	ns

The logistic regression modelling for the adverse outcome of the need for blood transfusion associated with admission ionised hypocalcaemia is shown in Table 8.

**Table 8:** Logistic regression for the adverse outcome of the need for blood transfusion in the first 24 hours associated with admission ionised hypocalcaemia for 45 paediatric major trauma patients

Parameter estimates	Variable	Estimate	Standard error	95% CI (profile likelihood)	
β0	Intercept	-2.588	2.294	-7.784 to 1.742	
β1	Blood transfusion within first 24 hours	2.435	1.516	-0.3896 to 5.959	
β2	Age	0.2341	0.1333	0.01415 to 0.5646	
β3	Gender	-0.8279	0.9852	-2.894 to 1.068	
β4	ISS	-0.02962	0.06369	-0.1804 to 0.08025	
β5	Pre-hospital fluids	-0.2382	1.461	-3.605 to 2.581	
β6	Acidosis (pH < 7.32)	-1.771	1.281	-4.958 to 0.4423	
Odds ratios	Variable	Estimate	95% CI (profile likelihood)		
β0	Intercept	0.07513	0.0004163 to 5.707		
β1	Blood transfusion within first 24 hours	11.41	0.6773 to 387.1		
β2	Age	1.264	1.014 to 1.759		
β3	Gender	0.437	0.05533 to 2.911		
β4	ISS	0.9708	0.8349 to 1.084		
β5	Pre-hospital fluids	0.788	0.02718 to 13.21		
β6	Acidosis (pH < 7.32)	0.1701	0.007026 to 1.556		
Sig. diff. than zero?	Variable	|Z|	P-value	P-value summary	
β0	Intercept	1.128	0.2591	ns	
β1	Blood transfusion within first 24 hours	1.606	0.1082	ns	
β2	Age	1.757	0.079	ns	
β3	Gender	0.8403	0.4007	ns	
β4	ISS	0.465	0.6419	ns	
β5	Pre-hospital fluids	0.163	0.8705	ns	
β6	Acidosis (pH < 7.32)	1.382	0.1669	ns	
Model diagnostics	Degrees of Freedom	AICc	Negative log likelihood value		
Intercept-only model	44	44.21	21.06		
Selected model	38	50.21	16.59		
Multicollinearity	Variable	VIF	R2 with other variables		
β0	Intercept				
β1	Blood transfusion within first 24 hours	1.246	0.1976		
β2	Age	1.148	0.1292		
β3	Gender	1.229	0.1863		
β4	ISS	1.574	0.3649		
β5	Pre-hospital fluids	1.522	0.3429		
β6	Acidosis (pH < 7.32)	1.31	0.2364		
Area under the ROC curve					
Area	0.8142				
Std. error	0.08742				
95% confidence interval	0.6429 to 0.9855				
P-value	0.0058				
Classification table	Predicted 0	Predicted 1	Total	% Correctly classified	
Observed 0	36	1	37	97.3	
Observed 1	6	2	8	25	
Total	42	3	45	84.44	
Negative predictive power (%)	85.71				
Positive predictive power (%)	66.67				
Classification cutoff	0.5				
Pseudo R squared					
Tjur's R squared	0.2114				
Cox-Snell's R squared	0.1802				
Hypothesis tests	Statistic	P-value	Null hypothesis	Reject null hypothesis?	P-value summary
Hosmer-Lemeshow	8.391	0.3963	Selected model is correct	No	ns
Log-likelihood ratio (G squared)	8.943	0.1768	Simpler (intercept-only) model is correct	No	ns

The logistic regression modelling for the adverse outcome of the need for vasoactive medications associated with admission ionised hypocalcaemia is shown in Table 9.

**Table 9:** Logistic regression for the adverse outcome of the need for vasoactive medications in the first 24 hours associated with admission ionised hypocalcaemia for 45 paediatric major trauma patients

Parameter estimates	Variable	Estimate	Standard error	95% CI (profile likelihood)	
β0	Intercept	-2.806	1.912	-7.154 to 0.7357	
β1	Need for vasoactive medications in the first 24 hours	0.9257	1.185	-1.490 to 3.409	
β2	Age	0.2222	0.118	0.02287 to 0.5066	
β3	Gender	-0.5554	0.9412	-2.509 to 1.276	
β4	ISS	-0.01928	0.05459	-0.1447 to 0.08015	
β5	Pre-hospital fluids	-1.139	1.541	-4.753 to 1.697	
β6	Acidosis (pH < 7.32)	-1.014	1.011	-3.224 to 0.8674	
Odds ratios	Variable	Estimate	95% CI (profile likelihood)		
β0	Intercept	0.06047	0.0007815 to 2.087		
β1	Need for vasoactive medications in the first 24 hours	2.524	0.2253 to 30.23		
β2	Age	1.249	1.023 to 1.660		
β3	Gender	0.5738	0.08134 to 3.583		
β4	ISS	0.9809	0.8653 to 1.083		
β5	Pre-hospital fluids	0.3201	0.008622 to 5.455		
β6	Acidosis (pH < 7.32)	0.3627	0.03981 to 2.381		
Sig. diff. than zero?	Variable	|Z|	P-value	P-value summary	
β0	Intercept	1.467	0.1423	ns	
β1	Need for vasoactive medications in the first 24 hours	0.7811	0.4348	ns	
β2	Age	1.883	0.0597	ns	
β3	Gender	0.5901	0.5551	ns	
β4	ISS	0.3532	0.7239	ns	
β5	Pre-hospital fluids	0.7393	0.4598	ns	
β6	Acidosis (pH < 7.32)	1.004	0.3156	ns	
Model diagnostics	Degrees of freedom	AICc	Negative log likelihood value		
Intercept-only model	44	44.21	21.06		
Selected model	38	52.45	17.71		
Multicollinearity	Variable	VIF	R2 with other variables		
β0	Intercept				
β1	Need for vasoactive medications in the first 24 hours	1.165	0.1417		
β2	Age	1.147	0.1279		
β3	Gender	1.219	0.1799		
β4	ISS	1.566	0.3613		
β5	Pre-hospital fluids	1.64	0.3902		
β6	Acidosis (pH < 7.32)	1.168	0.1442		
Area under the ROC curve					
Area	0.75				
Std. error	0.09374				
95% confidence interval	0.5663 to 0.9337				
P-value	0.028				
Classification table	Predicted 0	Predicted 1	Total	% Correctly classified	
Observed 0	37	0	37	100	
Observed 1	6	2	8	25	
Total	43	2	45	86.67	
Negative predictive power (%)	86.05				
Positive predictive power (%)	100				
Classification cutoff	0.5				
Pseudo R squared					
Tjur's R squared	0.1526				
Cox-Snell's R squared	0.1382				
Hypothesis tests	Statistic	P-value	Null hypothesis	Reject null hypothesis?	P-value summary
Hosmer-Lemeshow	7.132	0.5225	Selected model is correct	No	ns
Log-likelihood ratio (G squared)	6.695	0.3499	Simpler (intercept-only) model is correct	No	ns

The logistic regression modelling for the adverse outcome of the need for interventional radiology or an operation associated with the admission ionised hypocalcaemia is shown in Table 10.

**Table 10:** Logistic regression for the adverse outcome of the need for interventional radiology/operation in the first 24 hours associated with admission ionised hypocalcaemia for 45 paediatric major trauma patients

Parameter estimates	Variable	Estimate	Standard error	95% CI (profile likelihood)	
β0	Intercept	-2.979	2.064	-7.661 to 0.8705	
β1	Need for IR/operation in the first 24 hours	2.392	1.177	0.3122 to 5.072	
β2	Age	0.2385	0.1271	0.02659 to 0.5462	
β3	Gender	-0.3164	1.067	-2.511 to 1.834	
β4	ISS	-0.06416	0.06603	-0.2236 to 0.04919	
β5	Pre-hospital fluids	-0.8935	1.423	-4.234 to 1.802	
β6	Acidosis (pH < 7.32)	-1.496	1.123	-4.057 to 0.5330	
Odds ratios	Variable	Estimate	95% CI (profile likelihood)		
β0	Intercept	0.05087	0.0004708 to 2.388		
β1	Need for IR/operation in the first 24 hours	10.94	1.366 to 159.4		
β2	Age	1.269	1.027 to 1.727		
β3	Gender	0.7287	0.08116 to 6.258		
β4	ISS	0.9379	0.7996 to 1.050		
β5	Pre-hospital fluids	0.4092	0.01449 to 6.064		
β6	Acidosis (pH < 7.32)	0.2241	0.01731 to 1.704		
Sig. diff. than zero?	Variable	|Z|	P-value	P-value summary	
β0	Intercept	1.443	0.149	ns	
β1	Need for IR/operation in the first 24 hours	2.032	0.0422	*	
β2	Age	1.877	0.0605	ns	
β3	Gender	0.2965	0.7668	ns	
β4	ISS	0.9717	0.3312	ns	
β5	Pre-hospital fluids	0.628	0.53	ns	
β6	Acidosis (pH < 7.32)	1.332	0.1828	ns	
Model diagnostics	Degrees of freedom	AICc	Negative log likelihood value		
Intercept-only model	44	44.21	21.06		
Selected model	38	47.86	15.41		
Multicollinearity	Variable	VIF	R2 with other variables		
β0	Intercept				
β1	Need for IR/Operation in the first 24 hours	1.224	0.1828		
β2	Age	1.146	0.1277		
β3	Gender	1.22	0.1804		
β4	ISS	1.585	0.3692		
β5	Pre-hospital fluids	1.544	0.3523		
β6	Acidosis (pH < 7.32)	1.285	0.2219		
Area under the ROC curve					
Area	0.8716				
Std. error	0.05777				
95% confidence interval	0.7584 to 0.9848				
P-value	0.0011				
Classification table	Predicted 0	Predicted 1	Total	% correctly classified	
Observed 0	34	3	37	91.89	
Observed 1	6	2	8	25	
Total	40	5	45	80	
Negative predictive power (%)	85				
Positive predictive power (%)	40				
Classification cutoff	0.5				
Pseudo R squared					
Tjur's R squared	0.2494				
Cox-Snell's R squared	0.2219				
Hypothesis tests	Statistic	P-value	Null hypothesis	Reject null hypothesis?	P-value summary
Hosmer-Lemeshow	11.25	0.1879	Selected model is correct	No	ns
Log-likelihood ratio (G squared)	11.29	0.0798	Simpler (intercept-only) model is correct	No	ns

The logistic regression modelling for the adverse outcome of Glasgow Outcomes Scale ≤ 4 associated with admission ionised hypocalcaemia is shown in Table 11.

**Table 11:** Logistic regression for the adverse outcome of Glasgow Outcome Scale ≤ 4 in the first 24 hours associated with admission ionised hypocalcaemia for 45 paediatric major trauma patients

Parameter estimates	Variable	Estimate	Standard error	95% CI (profile likelihood)	
β0	Intercept	-3.01	1.99	-7.622 to 0.5941	
β1	GOS ≤ 4	0.8982	1.458	-2.433 to 3.855	
β2	Age	0.2518	0.1371	0.02859 to 0.5937	
β3	Gender	-0.5152	0.9429	-2.472 to 1.317	
β4	ISS	-0.02485	0.05506	-0.1522 to 0.07439	
β5	Pre-hospital fluids	-0.8049	1.434	-4.210 to 1.834	
β6	Acidosis (pH < 7.32)	-0.9986	0.9714	-3.051 to 0.8444	
Odds ratios	Variable	Estimate	95% CI (profile likelihood)		
β0	Intercept	0.04928	0.0004897 to 1.811		
β1	GOS ≤ 4	2.455	0.08779 to 47.22		
β2	Age	1.286	1.029 to 1.811		
β3	Gender	0.5974	0.08446 to 3.733		
β4	ISS	0.9755	0.8588 to 1.077		
β5	Pre-hospital fluids	0.4472	0.01485 to 6.258		
β6	Acidosis (pH < 7.32)	0.3684	0.04730 to 2.327		
Sig. diff. than zero?	Variable	|Z|	P-value	P-value summary	
β0	Intercept	1.513	0.1303	ns	
β1	GOS ≤ 4	0.6161	0.5378	ns	
β2	Age	1.836	0.0663	ns	
β3	Gender	0.5464	0.5848	ns	
β4	ISS	0.4514	0.6517	ns	
β5	Pre-hospital fluids	0.5611	0.5747	ns	
β6	Acidosis (pH < 7.32)	1.028	0.304	ns	
Model diagnostics	Degrees of freedom	AICc			
Intercept-only model	44	44.21			
Selected model	38	52.7			
Multicollinearity	Variable	VIF	R2 with other variables		
β0	Intercept				
β1	GOS ≤ 4	1.365	0.2675		
β2	Age	1.295	0.2279		
β3	Gender	1.224	0.1827		
β4	ISS	1.572	0.3637		
β5	Pre-hospital fluids	1.656	0.3961		
β6	Acidosis (pH < 7.32)	1.221	0.1809		
Area under the ROC curve					
Area	0.75				
Std. Error	0.09564				
95% confidence interval	0.5626 to 0.9374				
P-value	0.028				
Classification table	Predicted 0	Predicted 1	Total	% Correctly classified	
Observed 0	37	0	37	100	
Observed 1	6	2	8	25	
Total	43	2	45	86.67	
Negative predictive power (%)	86.05				
Positive predictive power (%)	100				
Classification cutoff	0.5				
Pseudo R squared					
Tjur's R squared	0.1575				
Cox-Snell's R squared	0.1335				
Hypothesis tests	Statistic	P-value	Null hypothesis	Reject null hypothesis?	P-value summary
Hosmer-Lemeshow	7.877	0.4456	Selected model is correct	No	ns
Log-likelihood ratio (G squared)	6.448	0.3749	Simpler (intercept-only) model is correct	No	ns

## References

[1] Goldstick JE, Cunningham RM, Carter PM (2022). Current causes of death in children and adolescents in the United States. N Engl J Med.

[2] Davis JS, Satahoo SS, Butler FK (2014). An analysis of prehospital deaths: who can we save?. J Trauma Acute Care Surg.

[3] Cottey L, Shanahan TA, Gronlund T, Whiting C, Sokunbi M, Carley SD, Smith JE (2023). Refreshing the emergency medicine research priorities. Emerg Med J.

[4] Ditzel RM Jr, Anderson JL, Eisenhart WJ, Rankin CJ, DeFeo DR, Oak S, Siegler J (2020). A review of transfusion- and trauma-induced hypocalcemia: Is it time to change the lethal triad to the lethal diamond?. J Trauma Acute Care Surg.

[5] Vasudeva M, Mathew JK, Groombridge C, Tee JW, Johnny CS, Maini A, Fitzgerald MC (2021). Hypocalcemia in trauma patients: a systematic review. J Trauma Acute Care Surg.

[6] Webster S, Todd S, Redhead J, Wright C (2016). Ionised calcium levels in major trauma patients who received blood in the Emergency Department. Emerg Med J.

[7] Hall C, Colbert C, Rice S, Dewey E, Schreiber M (2023). Hypocalcemia in trauma is determined by the number of units transfused, not whole blood versus component therapy. J Surg Res.

[8] Magnotti LJ, Bradburn EH, Webb DL (2011). Admission ionized calcium levels predict the need for multiple transfusions: a prospective study of 591 critically ill trauma patients. J Trauma.

[9] Vasudeva M, Mathew JK, Fitzgerald MC, Cheung Z, Mitra B (2020). Hypocalcaemia and traumatic coagulopathy: an observational analysis. Vox Sang.

[10] Ciaraglia A, Lumbard D, DeLeon M (2024). Retrospective analysis of the effects of hypocalcemia in severely injured trauma patients. Injury.

[11] Rushton TJ, Tian DH, Baron A, Hess JR, Burns B (2024). Hypocalcaemia upon arrival (HUA) in trauma patients who did and did not receive prehospital blood products: a systematic review and meta-analysis. Eur J Trauma Emerg Surg.

[12] Vettorello M, Altomare M, Spota A (2022). Early hypocalcemia in severe trauma: an independent risk factor for coagulopathy and massive transfusion. J Pers Med.

[13] Helsloot D, Fitzgerald M, Lefering R, Verelst S, Missant C (2023). Trauma-induced disturbances in ionized calcium levels correlate parabolically with coagulopathy, transfusion, and mortality: a multicentre cohort analysis from the TraumaRegister DGU(®). Crit Care.

[14] Imamoto T, Sawano M (2023). Effect of ionized calcium level on short-term prognosis in severe multiple trauma patients: a clinical study. Trauma Surg Acute Care Open.

[15] Dias CR, Leite HP, Nogueira PC, Brunow de Carvalho W (2013). Ionized hypocalcemia is an early event and is associated with organ dysfunction in children admitted to the intensive care unit. J Crit Care.

[16] Gimelraikh Y, Berant R, Stein M, Berzon B, Epstein D, Samuel N (2022). Early hypocalcemia in pediatric major trauma: a retrospective cohort study. Pediatr Emerg Care.

[17] Ciaraglia A, Lumbard D, Deschner B (2023). The effects of hypocalcemia in severely injured pediatric trauma patients. J Trauma Acute Care Surg.

[18] Epstein D, Ben Lulu H, Raz A, Bahouth H (2022). Admission hypocalcemia in pediatric major trauma patients-an uncommon phenomenon associated with an increased need for urgent blood transfusion. Transfusion.

[19] Cornelius BG, Clark D, Williams B (2021). A retrospective analysis of calcium levels in pediatric trauma patients. Int J Burns Trauma.

[20] Hibberd O, Price J, Thomas SH, Harris T, Barnard EB (2024). The incidence of admission ionised hypocalcaemia in paediatric major trauma-a systematic review and meta-analysis. PLoS One.

[21] Advanced Life Support Group (2023). Advanced paediatric life support: a practical approach to emergencies, 7th edition. https://www.alsg.org/home/course/view.php?id=394.

[22] Hibberd O, Price J, Laurent A, Agrawal S, Barnard E (2023). Paediatric major trauma: a Retrospective observational comparison of mortality in prehospital bypass and secondary transfer in the East of England. Cureus.

[23] Conner JR, Benavides LC, Shackelford SA (2021). Hypocalcemia in military casualties from point of injury to surgical teams in Afghanistan. Mil Med.

[24] Brennan L, Heal C, Brown S, Roland D, Rowland AG (2023). Time to change the reference ranges of children's physiological observations in emergency care? A prospective study. J Paediatr Child Health.

[25] Matthay ZA, Fields AT, Nunez-Garcia B, Patel MH, Cohen MJ, Callcut RA, Kornblith LZ (2020). Dynamic effects of calcium on in vivo and ex vivo platelet behavior after trauma. J Trauma Acute Care Surg.

[26] Morton S, Galea J, Uprichard J, Hudson A (2019). The practicalities and barriers of using TEG6s in code red traumas: an observational study in one London major trauma centre. CJEM.

[27] Rossaint R, Afshari A, Bouillon B (2023). The European guideline on management of major bleeding and coagulopathy following trauma: sixth edition. Crit Care.

[28] Chandler JM, Chan KS, Han R, Chao SD (2022). Mental health outcomes in pediatric trauma patients: a 10 year real world analysis using a large database approach. J Pediatr Surg.

[29] McMillan T, Wilson L, Ponsford J, Levin H, Teasdale G, Bond M (2016). The Glasgow Outcome Scale - 40 years of application and refinement. Nat Rev Neurol.

[30] Hsu CH, Couper K, Nix T, Drennan I, Reynolds J, Kleinman M, Berg KM (2023). Calcium during cardiac arrest: a systematic review. Resusc Plus.

